# The Present and Future of Whole Genome Sequencing (WGS) and Whole Metagenome Sequencing (WMS) for Surveillance of Antimicrobial Resistant Microorganisms and Antimicrobial Resistance Genes across the Food Chain

**DOI:** 10.3390/genes9050268

**Published:** 2018-05-22

**Authors:** Elena A. Oniciuc, Eleni Likotrafiti, Adrián Alvarez-Molina, Miguel Prieto, Jesús A. Santos, Avelino Alvarez-Ordóñez

**Affiliations:** 1 Faculty of Food Science and Engineering, Dunarea de Jos University of Galati, Galati 800008, Romania; elena.oniciuc@ugal.ro; 2 Laboratory of Food Microbiology, Department of Food Technology, Alexander Technological Educational Institute of Thessaloniki, Thessaloniki T.K. 57400, Greece; likotraf@food.teithe.gr; 3 Department of Food Hygiene and Technology and Institute of Food Science and Technology, Universidad de León, 24071 León, Spain; aalvm@unileon.es (A.A.-M.); miguel.prieto@unileon.es (M.P.); j.santos@unileon.es (J.A.S.)

**Keywords:** antimicrobial resistance, surveillance, foodborne pathogens, whole genome sequencing, metagenomics

## Abstract

Antimicrobial resistance (AMR) surveillance is a critical step within risk assessment schemes, as it is the basis for informing global strategies, monitoring the effectiveness of public health interventions, and detecting new trends and emerging threats linked to food. Surveillance of AMR is currently based on the isolation of indicator microorganisms and the phenotypic characterization of clinical, environmental and food strains isolated. However, this approach provides very limited information on the mechanisms driving AMR or on the presence or spread of AMR genes throughout the food chain. Whole-genome sequencing (WGS) of bacterial pathogens has shown potential for epidemiological surveillance, outbreak detection, and infection control. In addition, whole metagenome sequencing (WMS) allows for the culture-independent analysis of complex microbial communities, providing useful information on AMR genes occurrence. Both technologies can assist the tracking of AMR genes and mobile genetic elements, providing the necessary information for the implementation of quantitative risk assessments and allowing for the identification of hotspots and routes of transmission of AMR across the food chain. This review article summarizes the information currently available on the use of WGS and WMS for surveillance of AMR in foodborne pathogenic bacteria and food-related samples and discusses future needs that will have to be considered for the routine implementation of these next-generation sequencing methodologies with this aim. In particular, methodological constraints that impede the use at a global scale of these high-throughput sequencing (HTS) technologies are identified, and the standardization of methods and protocols is suggested as a measure to upgrade HTS-based AMR surveillance schemes.

## 1. Introduction

Antibiotics have been used in human and veterinary medicine for more than 70 years and have greatly contributed to tackling pathogenic bacteria and protecting human and animal health. However, the spread of antimicrobial resistant microorganisms in recent decades is emerging as a major challenge for mankind. Acquired resistance to some antimicrobials is now widespread to such an extent that their value for the treatment of certain life-threatening infections is being compromised. The high priority given to antimicrobial resistance (AMR) has led to the European Commission action plan against the rising threat of AMR [1] and the World Health Organization Global strategy for the containment of AMR [2]. Emergence and spread of AMR has usually been attributed to the continuous use of antibiotics as therapeutic drugs in human health care or in veterinary husbandry. However, there is a growing concern over the possibility of AMR transmission via the food chain with European strategic action plans on antibiotic resistance highlighting that AMR is a food safety issue [3].

Risk analysis schemes are used for the evaluation and communication of risks linked to foods and facilitate the decision making and the prioritization of measures for risk mitigation by national and international regulatory agencies. Success of risk assessment relies, among other factors, on adequate surveillance systems. Strengthening global AMR surveillance is critical, as it is the basis for informing global strategies, monitoring the effectiveness of public health interventions and detecting new trends and emerging threats.

Surveillance of AMR is currently being based on the isolation of indicator microorganisms and the phenotypic characterization of clinical, environmental and food strains isolated. This approach, which is based on the culture-dependent analysis of samples coupled to phenotypic and, sometimes, PCR-based genotypic tests of recovered isolates, can still be very valuable in AMR surveillance schemes and has been, and continues to be, extensively used in molecular epidemiology studies involving resistant strains from various foodborne pathogens, such as *Escherichia coli* [4], *Salmonella* spp. [5] and *Staphylococcus aureus* [6]. However, it does not provide complete information on the mechanisms driving AMR or on the presence or spread of AMR genes throughout the food chain. Several recent studies have demonstrated the potential of routine whole genome sequencing (WGS) of bacterial pathogens for epidemiological surveillance, outbreak detection, and infection control [7]. Metagenomics is also a powerful tool that allows for the culture-independent analysis of complex microbial communities, and has potential applications in AMR surveillance [8,9,10,11,12]. Indeed, it can provide access to all the genetic resources in a given environmental niche, which is essential for accessing the genomes of difficult-to-culture or non-cultivable microorganisms, and therefore could assist the tracking of AMR genes and mobile genetic elements, providing the necessary information to implement quantitative risk assessments to identify hotspots and routes of transmission of AMR across the food chain.

This review article summarizes the information currently available on the use of WGS and whole metagenome sequencing (WMS) for surveillance of AMR in foodborne pathogenic bacteria and food-related samples and discusses future needs that will have to be considered for the routine implementation of these next-generation sequencing methodologies with this aim or with the aim of identifying trends, neglected routes, and risk practices linked to AMR emergence or spread.

## 2. Whole Genome Sequencing of Foodborne Pathogens

The standardized analysis of bacterial susceptibility to antimicrobial agents relies on different phenotypic tests, such as broth micro- and macro-dilution methods or disk diffusion assays, which are later interpreted following different standardized guidelines, such as those established by the European Committee on Antimicrobial Susceptibility Testing (EUCAST) [13]. However, such techniques do not provide information on the genetic determinants conveying resistance or their association with mobile genetic elements, which may facilitate their global spread. The use of WGS can overcome those limitations [14], enabling not only the detection of resistant microorganisms and the identification of AMR determinants (and their genomic background) within surveillance schemes, but also the early detection of outbreaks or their epidemiological investigation [7,15].

**Figure 1:**
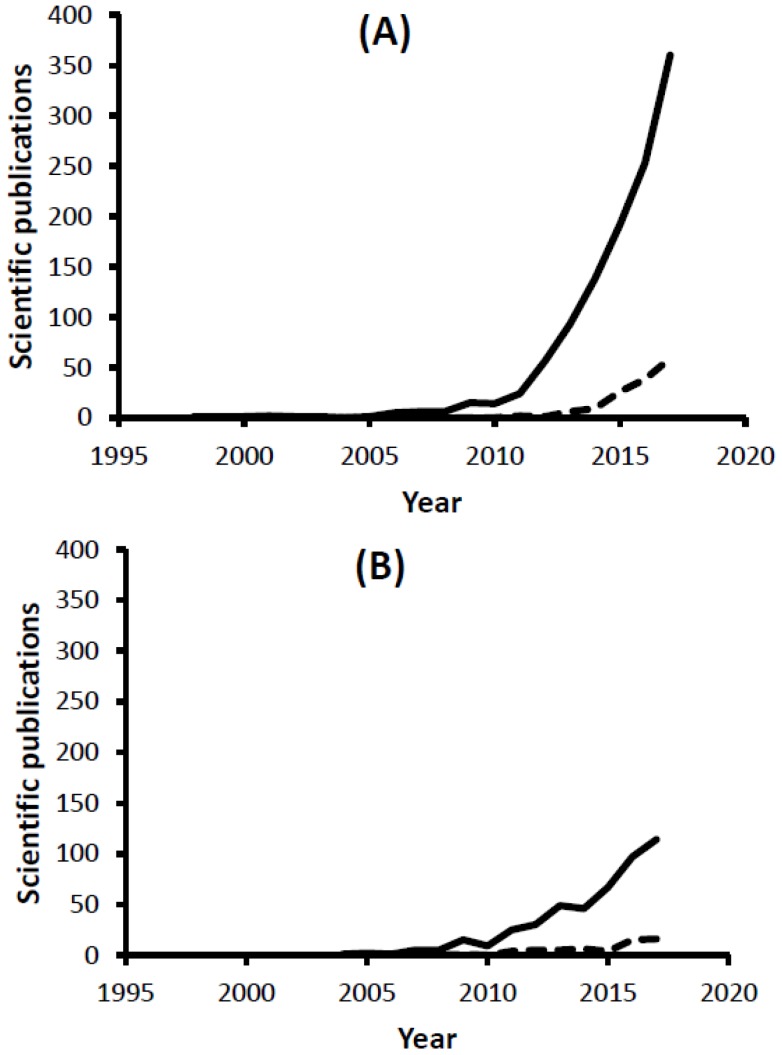
Number of scientific publications available on the literature on antimicrobial resistance (AMR) and whole genome sequencing (WGS) or whole metagenome sequencing (WMS). (**A**) Scientific publications obtained at [22] using the following search terms “whole genome sequencing AND (antimicrobial resistance OR antibiotic resistance)” (continuous line) or “whole genome sequencing AND food AND (antimicrobial resistance OR antibiotic resistance)” (discontinuous line); (**B**) Scientific publications obtained at [22] using the following search terms “metagenomic AND (antimicrobial resistance OR antibiotic resistance)” (continuous line) or “metagenomic AND food AND (antimicrobial resistance OR antibiotic resistance)” (discontinuous line).

WGS of microbial strains is becoming, due to the continuous decline in the associated costs, a powerful, very affordable and fast tool for microbial typing, which moreover achieves much higher resolution than traditional typing methods such as multilocus variable-number tandem repeat analysis (MLVA), pulsed field gel electrophoresis (PFGE), random amplified polymorphic DNA (RAPD), multiple-locus variable number tandem repeat (VNTR) and multilocus sequence typing (MLST) [16]. Indeed, a continuously increasing number of bacterial genomes are being published, offering an as yet understudied source of information in relation to AMR gene dynamics in clinical, environmental and food settings. It is nevertheless important to highlight that, despite the blossoming observed in genomic studies in relation to AMR since 2010, the application of WGS to survey AMR specifically in foods and food-related samples is not yet as extensive, representing 12.6% of all WGS-related peer-reviewed publications dealing with AMR (Figure 1A). Indeed, despite the recognized advantages of WGS, AMR surveillance in some countries still relies solely on phenotypic methods [14,17], while some authors recommend the combined use of phenotypic assays and techniques allowing the identification of genetic determinants of resistance for epidemiological surveillance purposes [18], and it has even been shown that phenotypic characterization of isolates can be improved through a detailed genetic characterization [19]. Indeed, some studies have demonstrated the potential of WGS-based genotyping of strains to predict their resistance phenotypic profiles. For instance, Neuert et al. analyzed 3491 non-typhoidal *Salmonella enterica* isolates to identify through WGS genes and chromosomal mutations responsible for phenotypic resistance, and compared the inferred genotypic AMR profiles with phenotypic susceptibilities determined for fifteen antimicrobials using EUCAST guidelines [20]. They found that only 0.17% of the isolate/antimicrobial combinations were discordant. Similarly, high concordance (99.74%) between phenotypic and predicted antimicrobial susceptibilities was observed by Zankari et al. when analyzing 200 *S. enterica* serovar Typhimurium, *E. coli*, *Enterococcus faecalis* and *Enterococcus faecium* strains isolated from Danish pigs [21]. Overall, these reports evidence the high capacity of WGS to be used for prediction of susceptibilities within surveillance schemes.

WGS-based AMR surveillance is already being conducted in the USA at state and local public health departments and universities within the framework of The National Antimicrobial Resistance Monitoring System (NARMS). This surveillance program tracks changes in antimicrobial susceptibility and characterizes AMR in enteric (intestinal) bacteria found in ill people (CDC), retail meats (FDA), and food animals (USDA), but, so far, it is only focused on four major foodborne bacteria, i.e., *Salmonella*, *Campylobacter*, *E. coli*, and *Enterococcus*. Other available initiatives include PulseNet, a network that compares bacterial DNA fingerprints obtained through PFGE, MLVA or WGS [23], or NCBI’s AMR finder, a project focused on foodborne pathogens and other organisms, which have so far curated an AMR gene database to which public health agencies in the USA and internationally can contribute by submitting WGS data [22]. However, a global initiative in the form of a centralized standard program for WGS-based AMR surveillance in isolates obtained from food, environment, and clinical samples has not yet been implemented at a global scale, and most countries still rely on phenotypic characterization of isolates recovered from food samples and clinical specimens and PCR-based diagnostics of AMR genes occurrence.

Most WGS applications for surveillance of AMR in foods available in the literature arise from studies conducted in the last five years and are focused on high-priority foodborne pathogens, such as *Salmonella* [24,25], *Campylobacter* spp. [26], Shiga toxin-producing *E. coli* [27], *Listeria monocytogenes* or *S. aureus* [28], although studies on bacteria that do not typically cause foodborne illness, such as *Enterococcus* or *Klebsiella*, have also been executed [29,30,31]. Initiatives have been aimed at discriminating resistant isolates coming from different sources [19,32], identifying AMR mechanisms or genetic determinants of resistance [33], defining and attributing infection sources in cases of food-related outbreaks caused by resistant microorganisms [34], or tracking the dissemination of AMR through the transfer of resistance genes [35]. The following paragraphs will focus on recent relevant reports performing in silico prediction of AMR using WGS-based approaches in four major well-characterized foodborne species, *Salmonella* spp., *Campylobacter* spp., *L. monocytogenes* and *E. coli*. Additional information on some of these, as well as other relevant reports monitoring AMR in food-related microorganisms through WGS, is presented in Table 1. Some of the reports discussed in the text use WGS mainly to address other scientific questions (e.g., source attribution, outbreak analysis), but indirectly provide information on AMR, which, in turn, may be further used to, for instance, facilitate the rapid selection of antibiotic-based therapies in clinical settings or execute AMR trends analyses.

### 2.1. Whole-Genome Sequencing of *Salmonella spp.*

*Salmonella* spp. is a major cause of foodborne gastroenteritis in humans, with 94,530 confirmed salmonellosis cases reported in 2016 in the European Union (EU) [72]. In decreasing order, the serovars most frequently involved in human cases of infection are *S. enterica* serovar Enteritidis, followed by *S. enterica* serovar Typhimurium, monophasic *S. enterica* serovar Typhimurium, *S. enterica* serovar Infantis and *S. enterica* serovar Derby [72]. Multidrug resistance in *Salmonella* spp. has been frequently reported [73,74], and several authors have associated the transmission of *Salmonella* spp. AMR with the consumption of raw or undercooked meat products [75,76], or the use of some biocides, such as triclosan, which has been reported to result in a decreased susceptibility to antibiotics due to mutations or overexpression of particular genes in *S. enterica* serovar Typhimurium [77].

WGS analysis of food-related *Salmonella* spp. isolates has become a first choice technique for use in salmonellosis outbreak investigations or epidemiological surveillance [24,78]. In a study conducted by Allard et al. [24], a set of different *S. enterica* serovar Montevideo strains coming from environmental, laboratory, clinical or food samples, and associated with repetitive contamination events, were analyzed through WGS in order to demonstrate the potential of this technology as a molecular typing technique. *S. enterica* serovar Montevideo is one of the most common *Salmonella* serovars associated with contaminated foods. Indeed, several foodborne outbreaks mentioned in the literature [24,79] have been attributed to this serovar through phenotypic and molecular detection (such as PFGE) assays, including outbreaks linked to pistachio, red and black pepper used in spiced meat production, raw sprouts, and different meat and dairy products [79,80].

Following WGS analysis and phenotypic characterization of 113 *S. enterica* serovar Heidelberg isolates from poultry meat (*n* = 44), poultry carcasses at the abattoir (*n* = 18) and humans (*n* = 51), Edirmanasinghe et al. [59] demonstrated the transmission of *S. enterica* serovar Heidelberg between livestock animals, retail poultry, and humans. They also showed that transmission of a common AMR plasmid (CMY-2), linked to microbial resistance against β-lactamic antibiotics, could occur among *S. enterica* serovar Heidelberg strains with different genetic backgrounds. Using WGS data, the *bla*_CMY-2_ gene was found to reside in two *S. enterica* serovar Heidelberg isolates on the largest contig (larger than 747 kb) corresponding to the chromosome, suggesting that the CMY-2 plasmid might have integrated into the chromosome in those two isolates. Moreover, the sequence analysis identified 10 plasmid subtypes that showed a high homology to the *S. enterica* serovar Kentucky pCVM29188_101 plasmid, also associated with the *bla*_CMY-2_ β-lactamase gene [59].

Tran-Dien et al. [63] performed a WGS study analyzing 288 *S. enterica* serovar Typhimurium isolates from clinical, animals, feed and food samples collected between 1911 and 1969 from 31 countries on four continents. This retrospective study highlighted that 4% of the analyzed isolates were resistant to ampicillin due to the carriage of various β-lactamase genes, including *bla*_TEM-1_, by different plasmids, including the virulence plasmid of *S. enterica* serovar Typhimurium. In addition, the eleven ampicillin-resistant *S. enterica* serovar Typhimurium genomes were clustered into three groups. The main group comprised seven ampicillin-resistant isolates collected from clinical, food and animal samples in France (1959–1969), which contained four types of β lactamase genes (*bla*_TEM-1A_, *bla*_TEM-1B_, *bla*_OXA-1_, and *bla*_OXA-2_). The second was composed of two ampicillin-resistant isolates collected from clinical samples in Tunisia (1960), while the third group comprised another two ampicillin-resistant isolates collected in France and Tunisia (1968). The authors showed that ampicillin resistance in *S. enterica* serovar Typhimurium emerged and has been transmitted over the years due to multiple independent acquisitions of *bla*_TEM_ gene-carrying plasmids by different bacterial populations [63].

Carroll et al. [33] compared different *S. enterica* serovars coming from dairy cattle and humans from an AMR point of view. Overall, AMR genes belonging to 42 different groups were detected in the assembled genomes by using Basic Local Alignment Search Tool (BLAST) [81] and Antibiotic Resistance Gene-ANNOTation [82], a tool that was created to detect existing and putative new AMR genes in bacterial genomes. Genes associated with resistance to penicillins were the most abundant, followed by the presence of genes of resistance to aminoglycosides (*aac(6)-Iaa*, *strA*, and *strB*), phenicols (*floR*), tetracyclins (*tet*(A) and *tet*(R)), cephalosporins (*CMY*) and sulphonamides (*sul2*) [33].

WGS has also been used to follow horizontal gene transfer events in *Salmonella* spp. In a study by Card et al. [83], a chicken gut model was used to monitor the transfer of a plasmid harboring multidrug resistance genes, including the extended-spectrum β-lactamase *bla*_CTX-M1_, from *S. enterica* serovar Typhimurium to the naturally resident commensal *E. coli* population. WGS of *E. coli* isolates from the chicken ceca prior to infection as compared to *E. coli* conjugants recovered from the chemostat system throughout the challenge test demonstrated that plasmid transfer occurred to seven *E. coli* sequence types at high rates, even in the absence of the selective pressure exerted by cefotaxime, with resistant *E. coli* strains being isolated within 3 days of incubation [83].

### 2.2. Whole-Genome Sequencing of *Campylobacter spp.*

Another foodborne pathogen that has been intensively studied by WGS is *Campylobacter* spp., a major cause of foodborne diarrhea in humans. *C. jejuni* and *C. coli* are the main species responsible for the majority of cases of campylobacteriosis [84]. Recent studies have indicated that *Campylobacter* isolates are frequently resistant to commonly used antimicrobials such as gentamicin and other aminoglycosides [85,86], tetracyclines, beta-lactams and fluoroquinolones [87]. *Campylobacter* isolates have frequently been isolated from raw meat, especially poultry, but also from raw milk, or even water [87]. As reported, in the European Union alone, the number of confirmed cases of campylobacteriosis has steadily increased in the last five years, with 246,307 confirmed human cases in 2016 [72].

Some studies have assessed the utility of WGS to understand the phylogenetic relationship of *Campylobacter* spp.-resistant clones and non-resistant isolates. In one study, Chen et al. [26], by sequencing two *C. coli* strains, showed the potential of WGS to find pTet-like plasmids not previously described, such as the plasmid pN29710-1, a self-transmissible plasmid carrying different AMR genes encoding resistance to gentamicin, kanamycin, tetracycline and streptothricin, which evolved from a pTet plasmid ancestor by insertion of multiple antibiotic resistance genes. Sequence analyses of such plasmids suggested that a phosphotransferase gene *aph(2*″*)-Ig* was responsible for gentamicin resistance, a fact that was further confirmed through heterologous expression studies [26].

Zhao et al. [38] analyzed 114 *Campylobacter* spp. (82 *C. coli* and 32 *C. jejuni*) strains recovered from a variety of sources—i.e., humans (40), retail meats (59) and cecal samples from food-production animals (15)—collected between 2000 and 2013 in the United States within the framework of the National Antimicrobial Resistance Monitoring System (NARMS) surveillance program. They identified 18 AMR genes (*tet(O)*, *bla*_OXA-61_, *catA*, *lnu(C)*, *aph(2*″*)-Ib*, *aph(2*″*)-Ic*, *aph(2*′*)-If*, *aph(2*″*)-Ig*, *aph(2*″*)-Ih*, *aac(6*′*)-Ie/aph(2*″*)-Ia*, *aac(6*′*)-Ie/aph(2*″*)-If*, *aac(6*′*)-Im*, *aadE*, *sat4*, *ant(6*′*)*, *aad9*, *aph(3*′*)-Ic*, *aph(3*′*)-IIIa*), and mutations in two house-keeping genes (*gyr*A and 23S rRNA), associated with resistance to different antibiotic families such as beta-lactams, tetracycline, chloramphenicol, lincomycin, and aminoglycosides. They also observed a high degree of correlation between phenotypic resistance to a given antibiotic and the presence of one or more corresponding resistance genes, demonstrating the effectiveness of WGS analyses to accurately predict resistant phenotypes in *Campylobacter* spp.

A recent study conducted by Yao et al. [84] on 607 *Campylobacter* isolates from cecal contents of poultry and swine fecal samples in different provinces of China identified *aph(2*″*)-If* as the dominant gentamicin resistance determinant in *Campylobacter*. WGS analyses demonstrated that *aph(2*″*)-If* was located on a chromosomal segment inserted between two conserved genes, *Cj0299* and *panB*, and suggested that both regional expansion of a particular clone and horizontal transmission were involved in the dissemination of the *aph(2*″*)-If* gene in *Campylobacter*.

### 2.3. Whole-Genome Sequencing of *Listeria monocytogenes*

*L. monocytogenes* is a zoonotic agent mainly transmitted through consumption of contaminated foods, such as milk and dairy products, different types of meat and meat products, fish products and vegetables like fresh lettuce, radishes, or cabbage [88,89]. In addition, it has the ability to colonize food production facilities and persist in them for years, with its persistence being sometimes attributed to its ability to develop resistance to disinfectants and other antimicrobials [53,89,90].

Several authors have addressed the utility of WGS to investigate the genetic basis underlying the virulence and adaptability of this foodborne pathogen [52], particularly in the context of large listeriosis outbreaks. Kwong et al. [91] analyzed a total of 520 *L. monocytogenes* isolates from food, environmental or clinical samples at the Microbiological Diagnostic Unit Public Health Laboratory of Australia, and identified distinct nested clusters within groups of isolates that were otherwise indistinguishable by other currently available typing methods. The potential of WGS for inferring linkage to point source outbreaks was also shown. In fact, a high-resolution scheme identified isolates that were epidemiologically linked, like, for example, a mother-to-baby pair belonging to sequence type (ST) 3, which also clustered isolates coming from food industry sampling. They therefore demonstrated the high potential of WGS as a routine surveillance tool, allowing the detection of outbreaks in real time. A similar approach was followed by Hyden et al. [92], who used WGS of *L. monocytogenes* for serogroup determination. WGS was also used to characterize *L. monocytogenes* clones associated with a listeriosis outbreak occurring in Austria and Germany between mid-2011 and 2013 [93]. These authors studied human and food isolates associated with seven human cases of listeriosis. The results confirmed that 4 out of 5 Austrian cases were linked to Austrian food producers. In addition, the epidemiological results regarding food isolates differentiated the three cases emerging in 2011 from the other four cases, linked to the 2012–2013 outbreak. Chen et al. [94] studied, through WGS, *L. monocytogenes* isolates from food, environmental and clinical samples associated with a listeriosis outbreak occurring in 2015 in the United States due to the consumption of different ice cream products. WGS analyses performed by these authors clustered together outbreak-associated isolates that exhibited multiple PFGE profiles but differed in a few single nucleotide polymorphisms (SNPs), making it possible to differentiate them from epidemiologically unrelated isolates that exhibited undistinguishable PFGE profiles, evidencing the benefits offered by WGS in outbreak investigation.

Apart from all these studies demonstrating the promise of WGS as a *L. monocytogenes* surveillance tool, this technique has also been employed to characterize AMR in *L. monocytogenes* food and clinical isolates. Ortiz et al. [53] used WGS to reveal determinants associated with *L. monocytogenes* persistence in food processing industries, and to evaluate whether there is any connection between the use of particular antimicrobials (disinfectants) and persistence status. For this aim, two meat-processing plants in Spain were investigated, a pork processing facility that manufactured fresh and cured meat products and also exported them to the United States (plant A), and a second facility, newly built, that received such products from plant A (plant B) [53,95]. These authors isolated in their study several benzalkonium chloride (BAC)-resistant isolates, corresponding to PFGE types S1 and S10-1 from plant A and S2-2, S2-3, and S10-3 from plant B, which were studied through WGS. Large genomic differences among BAC-resistant isolates were observed, with S1 PFGE type (ST31 by MLST) isolates belonging to a low-virulence type, due to the presence of certain mutations in the *inlA* and *prfA* genes, and containing the stress survival islet 1 (SSI-1), which may facilitate environmental survival. The remaining four BAC-resistant isolates belonged to ST121 and were equipped with different AMR and stress resistance genes. The genome features revealed that those ST121 strains harbored a transposon Tn*6188* responsible for increased tolerance against quaternary ammonium compounds (QAC), a cadmium resistance transposon (Tn*5422*) and *clpL* genes, which have been shown to be involved in stress response.

Fox et al. [96] sequenced two *L. monocytogenes* isolates indistinguishable from one another via PFGE. Both genomes contained genes encoding for non-specific multidrug efflux pumps, associated with an increased resistance to tetracycline, lincomycin, quinolone, and beta-lactams but also many non-specific heavy metal resistance genes. These authors also demonstrated the greater discriminatory power of WGS in comparison with other typing techniques such as PFGE or MLST, as the genomes shared only 99% nucleotide sequence identity, while both strains were indistinguishable through PFGE.

Another recent study carried out a comparative genomic analysis of two Malaysian *L. monocytogenes* strains isolated from fried fish (LM115) and salad (LM41) [52]. These authors identified virulence and AMR genes by using the Virulence Factors of Pathogenic Bacteria Database (VFBD) and the Resistance Gene Identifier (RID) of the Comprehensive Antibiotic Resistance Database (CARD). Both studied strains carried different antibiotic and efflux pump related genes which may confer resistance against lincomycin, erythromycin, fosfomycin, quinolones, tetracycline, β-lactams, and macrolides, including *mec*C, *lmrB*, *mrsA*, *fosX*, *Ide* and *mdrL*.

More recently, Wilson et al. [97] predicted the AMR profile of 100 *L. monocytogenes* isolates coming from dairy (52), meat (2), vegetables (2), food (2), seafood (1) and dairy farm environment (1) samples, recovered from Australian food production chains between 1988 and 2016, by using their draft genomes. The genetic markers of AMR identified included fosfomycin resistance (*fosX*) and lincomycin resistance (*lmrB*) genes. Only one isolate (Lm16-001) harbored an erythromycin resistance gene, *ermB*, which could be correlated with the phenotypic observations on erythromycin resistance (minimum inhibitory concentration >256 mg/L).

### 2.4. Whole-Genome Sequencing of *Escherichia coli*

Finally, another pathogen that has received attention in recent decades is *E. coli*, a microorganism predominantly associated with the consumption of meat and meat products, which is also part of the endogenous microbiota of humans and animals [43]. *E. coli* has been identified as a reservoir of AMR genes in the food chain linked to the widespread use of diverse antimicrobials in farm animals. Hussain et al. [43] conducted a study in which broiler and free-range chicken outlets from different cities belonging to different states of India were sampled, assessing the antimicrobial susceptibility patterns of recovered *E. coli* isolates. These authors also analyzed through WGS a total of 168 *E. coli* isolates and compared the genetic relationship of these isolates with strains from human *E. coli* pathotypes. They confirmed two emergent *E. coli* human pathogenic lineages [43]. Of particular interest are Shiga toxin-producing *E. coli* (STEC) strains, which are capable of carrying novel antibiotic resistance plasmids [46]. These authors analyzed twenty-six *E. coli* strains (twenty-two STEC and four non-STEC strains) through WGS, isolated either from clinical, animal (pigs, rabbits, goats and horses) or environmental samples, and identified 39 new plasmids. Two of these plasmids carried six genes linked to resistance to certain classes of antibiotics such as carbapenems, cephalosporins, aminoglycosides, penicillins, chloramphenicol, tetracyclines and sulphonamides. In addition, two other novel IncHI2 plasmids known to play a role in the acquisition of AMR were also identified [46]. Similarly, He et al. [48] reported the presence of a *bla*_NDM-5_ gene located on a pNDM-MGR194-like plasmid in three of the *E. coli* isolates studied through WGS (from dairy cattle in the Chinese Jiangsu province). Interestingly, in one particular isolate, the *bla*_NDM-5_ gene coexisted with the *mcr-1* colistin resistance gene [48].

## 3. Whole Metagenome Sequencing

**Figure 2:**
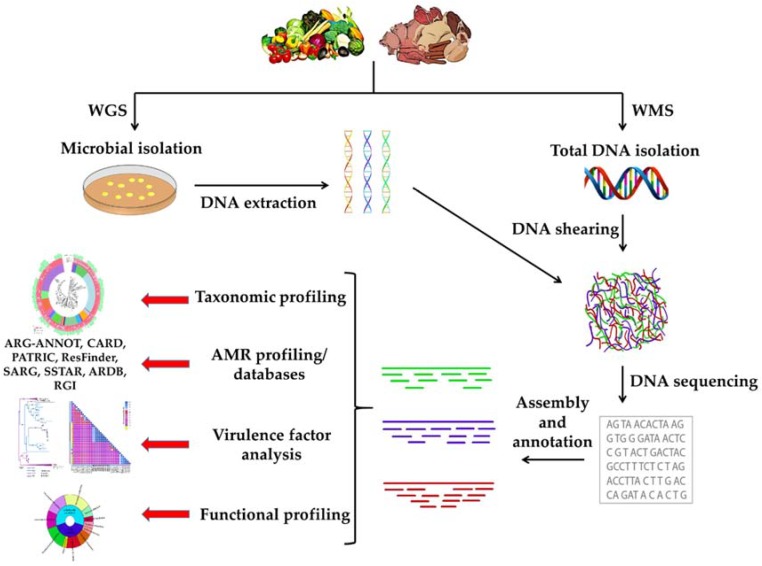
Schematic workflow of the approaches that may be followed when implementing WGS or WMS methodologies for AMR surveillance in foods. ARG-ANNOT: Antibiotic Resistance gene-ANNOTation; CARD: Comprehensive Antibiotic Resistance Database; PATRIC: Pathosystems Resource Integration Center; ResFinder: Antimicrobial resistance genes and/or chromosomal mutations database; SARG: Structured Antibiotic Resistance Genes Reference Database; SSTAR: Sequence Search Tool for Antimicrobial Resistance; ARDB: Antibiotic Resistance Genes Database; and RGI: Resistance Gene Identifier, respectively.

Metagenomic approaches are culture-independent alternatives for direct characterization of the microbiota of food, water, fecal, soil, or environmental samples, among others [98]. The term metagenome sequencing is used for two different high-throughput sequencing (HTS) approaches: amplicon sequencing and WMS [99]. In amplicon sequencing, marker genes, such as 16S rRNA or 18S rRNA genes, are amplified by PCR, from DNA extracted from a microbial community, and they are subsequently subjected to direct sequencing and aligned against a reference database in order to determine the taxonomic composition of the sample. Microbial identification is usually achieved down to the genus level. On the other hand, WMS (Figure 2) involves the fragmentation and subsequent sequencing, assembly and annotation of total genomic DNA isolated from a given sample (e.g., a food), and makes it possible to gain information on its entire (prokaryotic and eukaryotic) gene content [100]. WMS outcompetes targeted amplicon sequencing in that it provides species- or even strain-level identification [101], and, in addition, it offers insights into the metabolic, virulence or resistance potential of the studied microbial communities, while amplicon sequencing mainly provides information on population structure and only makes it possible to obtain very general predictions on the metabolic potential of the microbial community [99]. In the last decade, a blooming of microbial ecology studies based on WMS has occurred, aimed at gaining a deeper understanding of complex microbial communities in diverse environmental settings. Food and food-related samples are also being studied using this HTS approach, although the number of available scientific publications is still quite limited as compared to other ecosystems, representing 13% of all WMS-related peer-reviewed publications dealing with AMR (Figure 1B). Some examples of WMS applications in the Food Science field are the detection of foodborne pathogens in food, the investigation of outbreaks or the transmission of microorganisms through food production chains, the monitoring of microbial successions throughout fermentation of foods, or the identification of changes in microbial populations leading to food defects or spoilage [99]. Some of the reports discussed in the following lines deal only marginally with AMR, with their main focus being the characterization of the microbiota and the study of the microbial successions in particular foodstuffs and food-related samples. Nevertheless, it is important to note that, even in those cases where not much attention has been given to AMR, raw reads are freely available and can be downloaded and analyzed, even by other research groups, therefore representing an invaluable source of data on the occurrence of AMR genes in a wide range of foods and samples.

### 3.1. Whole Metagenome Sequencing in Food Ecosystems

WMS can be a powerful tool for monitoring foodborne pathogens throughout the food supply chain. Specific virulence factors or resistance markers can then be detected by using this technology, which enables, for example, the differentiation of the various pathovars of *E. coli* from other non-pathogenic strains.

WMS was used experimentally by Leonard et al. [102] to detect Shiga toxin-producing *E. coli* in spiked spinach samples. These authors showed that WMS provided adequate sequencing depth down to strain level, allowing the detection of key virulence determinants, even in samples inoculated with low numbers of the target organism, such as ≈10 CFU/100 g, which evidenced the potential of the technique for microbiological safety surveillance of fresh produce. In a follow-up study by the same research team [103], spinach was spiked with different strains of Shiga toxin-producing *E. coli* at a low concentration of 0.1 CFU/g and then subjected to WMS analyses, which successfully achieved strain level identification of the pathogenic strains despite the presence of indigenous *E. coli* strains. In a survey on the safety of nunu, a traditional Ghanaian fermented milk product, Walsh et al. [10] applied three short-read alignment-based bioinformatics methods (MetaMLST, PanPhlAn, and StrainPhlAn) in order to identify pathogenic strains. They detected putative pathogenic *E. coli* and *Klebsiella pneumoniae* strains in some nunu samples, indicating fecal contamination, poor hygiene in the production process and a potential hazard to the health of consumers.

Apart from members of the *Enterobacteriaceae*, Gram-positive pathogens have also been detected using WMS in foodstuffs. Different strains of *L. monocytogenes* were detected and characterized through WMS from samples of ice cream linked to a listeriosis outbreak by Ottesen et al. [104]. The authors found three slightly variable *L. monocytogenes* genomes and proposed that consensus draft genomes of *L. monocytogenes* can be produced from metagenomics sequencing data after 48 h of enrichment, which could potentially be used to trace back outbreak-associated strains upon validation of this approach.

Yang et al. [105] used WMS to detect pathogens at species level and their virulence factors, and to observe shifts in pathogen population along the beef production chain. They reported a dramatic reduction of the relative abundance of all pathogenic and non-pathogenic bacteria from the feedlot to the final meat products, but relative proportions of some pathogenic species increased in the remaining microbiota present in the end products. It was hypothesized that this was due to the lack of competition from other bacteria or to the ability of the pathogens in question to survive the antimicrobial interventions often used in beef processing—e.g., physical interventions (knife trimming, steam-vacuuming), use of organic acids and oxidizing antimicrobials, non-thermal interventions (UV, ozonated water), or thermal interventions (hot-water).

WMS is also a valuable tool for the investigation of food poisoning cases and for epidemiological studies, as it can be applied to the detection and characterization of foodborne pathogens in clinical samples and food. Huang et al. [106] demonstrated, when analyzing stool samples using WMS, that *S. enterica* serovar Heidelberg was the pathogen responsible for two foodborne outbreaks in different states (Alabama and Colorado), thus confirming the results of culture-dependent techniques. In addition, the authors could detect significant shifts in the gut microbiome of patients affected by the infection, as well as overgrowth of commensal *E. coli* and underlying co-infections with *S. aureus*, proving that metagenomics can offer very helpful information in foodborne outbreak cases. In a large outbreak of food poisoning caused by Shiga-toxigenic *E. coli* (STEC) O104:H4 in Germany, WMS was used by Loman et al. [107] to identify the outbreak strain from fecal samples obtained from 45 patients. These authors developed a de novo assembly approach to obtain a draft genome of the outbreak strain and detected sequences from the Shiga-toxin genes in 27 out of 40 STEC-positive samples. Furthermore, sequences from *Clostridium difficile*, *Campylobacter jejuni*, *Campylobacter concisus* and *S. enterica* were also recovered in their analyses.

WMS results have been also used to improve current culture-based detection techniques for both culturable and non-culturable pathogens in routine microbiology laboratories through the modification of enrichments to avoid the interference of background microbiota in the recovery of the desired pathogens or by reducing/eliminating the need for enrichment [108]. More specifically, Ottesen et al. [109] observed, using WMS, that the enrichment media widely used for *Salmonella* isolation allowed *Paenibacillus* spp. to outcompete and even inactivate *Salmonella* from the tomato phyllosphere, which suggests that alternative enrichment media should be used for *Salmonella* detection/isolation. Similarly, Jarvis et al. [110], carrying out microbiological analyses in cilantro, described that a 24-h nonselective pre-enrichment step favors the growth of Gram-positive Firmicutes, rather than Proteobacteria such as *Salmonella*, and therefore suggested that other pre-enrichment media should be used and recommended that control spike studies be undertaken in order to predict shifts in *Salmonella* abundance during enrichment.

In addition to food safety issues, WMS is extremely useful for investigating spoilage events and to characterize the shifts in microbial populations playing a role in the production of fermented foods. For example, WMS was used by Quigley et al. [111] to identify the uncultured bacterium *Thermus thermophilus* as the cause of cheese pinking spoilage, which would not have been possible using culture-based methods. Similarly, Hong et al. [112] analyzed rice wine samples with WMS to determine the differences in microbial populations between good and poor quality wine. Apart from the bacteria themselves, this method also detected the genes responsible for undesirable metabolic products.

Fermented milk products are very important food sources in many parts of the world and can contain a diverse and changing microbial flora. The beverage kefir is made from kefir grains comprising a complex mixed community of yeasts and bacteria. Nalbantoglu et al. [113] used WMS to show that *Lactobacillus kefiranofaciens*, *Lactobacillus buchneri* and *Lactobacillus helveticus* were the dominant species, while Walsh et al. [114] observed a succession in which the initially-dominant *L. kefiranofaciens* was overtaken by *Leuconostoc mesenteroides* in the latter stages of the fermentation. Microbial succession in a surface-ripened cheese was also studied using WMS and it was reported that yeasts and *Lactococcus lactis*, which were dominant at the start of ripening, were superseded by *Corynebacterium casei* as the ripening progressed [115]. WMS was also used to determine that *Lactobacillus*, *Leuconostoc* and *Weissella* were the predominant genera in a Mexican Cotija cheese. The contribution of different bacteria to the flavor of the cheese was also elucidated [116]. A similar study was performed on the Korean fermented vegetable product kimchi by Jung et al. [117], in which the dominant fermentative bacteria were identified. The authors also detected a large number of phage DNA sequences, suggesting that the fermentation process may have been affected by phage infection of the bacterial culture. Interestingly, phages could serve as vehicles for AMR spread through transduction events [118].

### 3.2. Antimicrobial Resistance Surveillance through Metagenomics

Although the use of WMS for screening AMR genes in food or clinical samples within surveillance schemes is very promising, current hurdles and difficulties have to be overcome in order to routinely implement this technology in official control programs. One of the major problems of predicting AMR genes from WMS reads is the databases used, which produce a high rate of false negatives based on the ‘best hits’ of sequence searches. To address this problem, research is currently under way and two new models have been recently developed to offer more accurate AMR annotation for both short read sequences and full gene length sequences [119]. Moreover, a study by Fitzpatrick and Walsh [120] analyzing the distribution and relative abundances of resistance genes in human, animal, water, soil, plant and insect metagenomes concluded that, for WMS analysis, limits of detection should be established in order to guarantee detection of even rare AMR genes in complex and diverse microbiome populations.

So far, WMS has scarcely been used to investigate AMR in foods, despite the recognized role of the food production chain in the spread of antimicrobial resistant microorganisms. Nevertheless, some studies have followed functional metagenomics approaches to detect novel and highly divergent AMR genes in the food chain reservoir. To achieve this, total community genomic DNA is cloned into a vector and transformed into a susceptible expression host, which is then cultured onto selective media to identify AMR phenotypes. Subsequent sequencing and annotation of the vector conveying resistance allows the identification of AMR genes [108]. For instance, functional metagenomics were used for the analysis of tetracycline resistant bacteria in a traditional raw-milk, blue-veined Spanish cheese. The shift of microbial populations during cheese ripening could account for the observed evolution of tetracycline resistance gene types, which are frequently located in plasmids, posing a risk for horizontal transfer [8]. Similarly, Devirgiliis et al. [121] constructed a metagenomic library containing microbial DNA extracted from mozzarella cheese and observed ampicillin- and kanamycin-resistant clones originating from *Streptococcus salivarius* subsp. *thermophilus* and *L. helveticus* genomes. Additionally, Berman and Riley [122] created metagenomic plasmid libraries from microbiota isolated from retail spinach in order to detect novel AMR genes. They identified novel DNA sequences conferring antibiotic resistance, which were linked to commensal or saprophytic bacteria, and concluded that food saprophytes could be a reservoir for new drug-resistance determinants in human pathogens.

Specifically dealing with WMS, the microbiota of milk samples from cattle with subclinical mastitis was investigated using WMS by Bhatt et al. [123]. In pure-bred Krankej and Gir cattle, *E. coli* was the predominant species followed by *Pseudomonas aeruginosa*. In milk from cross-bred cattle, *S. aureus* was present in the highest numbers, followed by *P. aeruginosa*. Several genes conferring resistance to fluoroquinolones and methicillin were detected, which, together with other antibiotic resistance sequences, indicated potential multidrug resistance within the microbial population [123]. Naik et al. [124] carried out a similar investigation into the microflora of five marine fish species. *Photobacterium* and *Vibrio* were the most predominant fish and human pathogens detected followed by *Shewanella, Acinetobacter*, *Psychrobacter* and *Flavobacterium*. Multiple antibiotic resistance was common and many different AMR genes were detected, including some on highly mobile plasmids and Class I integrons, which could be transferred to other bacteria in the food chain and in the gut.

AMR has been extensively studied and associated with the use of antibiotics in animal husbandry. AMR genes can spread from this reservoir to the environment and from there to humans, either directly or indirectly via the food chain [125]. The resistome of dairy and beef production effluents in North America was studied through WMS by Noyes et al. [126]. These authors revealed 34 AMR genes, mostly associated with tetracycline resistance, within soil, manure and wastewater samples, from feedlot, ranch and dairy operations. However, they did not identify any AMR determinants in the final beef products, suggesting that interventions in slaughterhouses could reduce the risk of AMR transmission. Pitta et al. [127], using a WMS approach, identified 18 AMR gene classes in dairy agroecosystems, with the most abundant AMR genes being classified as multidrug transporters, followed by vancomycin, tetracycline, bacitracin, and β-lactam resistance determinants.

### 3.3. Advantages and Disadvantages of Whole Metagenome Sequencing in Antimicrobial Resistance Surveillance across the Food Production Chain

WMS provides valuable information on microbial communities and how they interact in foods such as cheese [115] or kimchi [117], as well as on microbial dynamics in some particular food production chains [105], and, therefore, it has a tremendous potential as a food analytical technique, with various possible applications. WMS can identify previously unknown or emergent microbes [128]; it can also be used in order to detect food fraud, food mislabeling, species fraud [129] and to screen food products and ingredients for unauthorized genetically modified organisms (GMOs) in order to enhance public surveillance and help law enforcement agencies and food regulators; it could even be a useful tool for the authentication of products with Protected Designation of Origin (P.D.O.) status, such as cheese, wine and other fermented foods, based on microbiome fingerprinting, in order to prevent mis-selling and to protect producers [130,131]. Specifically talking about AMR, WMS allows the detection of AMR genes in complex food samples, providing information on their prevalence, distribution and possible routes of transmission in the food production chain, which may facilitate the design of interventions aimed at reducing AMR risk [127,132]. One of the many advantages of WMS in this regard includes the simultaneous detection of phage and bacterial DNA, which would have been impossible with other conventional molecular techniques [133], which may facilitate identification of horizontal gene transfer events.

Nevertheless, for WMS to be exploited in AMR surveillance and public health monitoring schemes, bioinformatics tools and databases would need to be adapted and continuously updated in order to understand resistance mechanisms, characterize the possible AMR threat and predict and identify known and novel AMR genes from foods and food production environments. According to Martínez et al. [134], AMR genes found in metagenomics studies should be ranked according to the risk they pose to human health. This could help eliminate the ‘noise’ observed in bioinformatics databases when new data is added without functional annotation. WMS analyses create vast amounts of data and require specialized bioinformatics expertise. Despite the continuous decline in sequencing costs, WMS is still quite costly as a technique and can take days/weeks before curated results are obtained, which renders it not very usable at the moment for food safety purposes in routine screening laboratories [135]. There exists a need for more reliable databases that can be used as reference to interpret the biological data obtained [136]. In addition, both wet lab and bioinformatics methodologies must be standardized in order to allow for the comparison of results among labs and samples. It has been shown in a number of occasions that the wet lab methodology (sampling methods, DNA isolation methods, enrichment vs non-enrichment before DNA isolation, etc.) and the selection of bioinformatics tools have a huge impact on the results obtained when analyzing a given sample through WMS [66,137]. Therefore, the validation of standard methods for WMS of foods would undoubtedly improve the reliability of the analyses performed. In some other fields, standard methods for WMS of samples are already available (e.g., stool samples—human microbiome studies) [138]; however, in the Food Science field, this has not been fully addressed yet. Another issue is that with WMS there is no distinction between the viable and non-viable bacteria of a microbiome. Thus, when DNA from a particular microorganism is detected in food matrices, it can come from viable cells, viable but not culturable (VBNC) cells, or from dead or inactivated cells, especially since most foods undergo specific processing steps which kill bacteria, while DNA can still be detected in the food sample [139]. However, some measures can be taken to reduce this particular problem. Erkus et al. [140] reported that treating Gouda cheese with propidium monoazide (PMA) prior to DNA extraction enhanced the amplification of the intact DNA while inhibiting the amplification of DNA from membrane-damaged cells. Interferences due to the presence of genetic material of non-microbial origin is also a problem in many sample types. Noyes et al. [132], when trying to assess AMR determinants in final beef products through WMS, found that the vast majority of the reads from meat originated from the genomes of the slaughtered animals. Furthermore, when WMS is used to detect virulence or AMR genes, it is difficult to predict if they belong to a specific pathogen or to the background microbiota, hence more advanced bioinformatics tools are necessary for secondary analysis. Indeed, one of the challenges WMS faces is the difficulty in attributing the identified resistance genes to specific strains. However, recent advances demonstrating the possibility of carrying out assembly of microbial genomes from WMS data using Hi-C or other techniques [141] could allow the attribution of particular AMR genes to specific bacteria, which will allow the risk ranking of the AMR genes present in a given sample. In fact, AMR genes present in non-pathogenic strains would be less hazardous, unless located in mobile genetic elements, which could facilitate its transfer to pathogenic bacteria.

## 4. Conclusions and Futures Prospects

Despite their huge potential, implementation of WGS and WMS as routine laboratory tools for surveillance of foodborne pathogens and AMR from foods is still in its infancy. Only a small number of countries and laboratories have acquired the resources and expertise to establish WGS approaches in epidemiology and public health surveillance [142]. Towards this goal, the Food and Drug Administration (FDA) has developed Genome Trakr, a database for the genomes of foodborne pathogens, aiming to help scientists to identify the food source of an outbreak [143].

As WGS and WMS technologies become more widely adopted, the key challenges of generating representative data sets and the development of bioinformatics tools to manage and interpret the data become increasingly pertinent. In the last decades, a vast number of resistant microorganisms and AMR genes have been described in multiple environments. Nevertheless, as mentioned above, it is necessary to consider the risks for human health associated with each of the resistance determinants predicted to confer antibiotic resistance in order to select appropriate indicators to be included into a risk assessment scheme using Next Generation Sequencing methodologies. Martínez et al. [134] and Berendonk et al. [144] have recently proposed a framework for the risk ranking of antibiotic resistant bacteria and AMR genes. They advise considering, among other factors, whether the resistance determinants are already known to contribute to the failure of antibiotic treatments, whether they have previously been reported to reside on mobile genetic elements hosted by human pathogens or by non-pathogenic bacteria, and whether they confer antibiotic resistance to clinically relevant antibiotics, new antibiotics in Phase I, II or III development, antibiotics just starting clinical use or no longer widely used. They also identify some bacterial species and candidate genes frequently occurring in environmental settings subjected to human activities as putative indicators of antibiotic resistance.

Culture-independent data generated through WGS and WMS can be analyzed to determine the occurrence and distribution of AMR determinants in a range of environments, including foods, food-related environments and clinical specimens. In addition, this information can be integrated with metadata gathered from food or clinical samples allowing for the implementation of quantitative risk assessment frameworks modelling resistance determinants occurrence and distribution. Predictive modelling of AMR genes distribution in environmental niches has been for instance recently implemented by Amos et al. [145], who used data on class 1 integron prevalence in the Thames river catchment (London, UK) together with geospatial and chemical metadata to produce a predictive model attributing sources of AMR. Similar approaches can be implemented to facilitate the identification of risky practices, sources and hotspots of AMR along the food chain, through WGS and WMS.

Despite the demonstrated potential of WGS and WMS tools within surveillance programs, their routine use does require its transformation into cheaper, user-friendly approaches that could be used on-site by personnel not specialized in big data management [146]. In this regard, prototypes of some miniaturized sequencers have recently become available and have demonstrated potential to be used on-site, generating results in real time [147]. In addition, the availability of open access AMR databases specially dedicated to food microbiology ecosystems, updated at real time and freely accessible, can pave the way for a more wide exploitation of these molecular tools [148].

## Figures and Tables

**Table 1:** Main research studies published in recent years applying whole genome sequencing (WGS) to characterize antimicrobial resistance (AMR) in foodborne bacteria.

Reference	Microbial Species	Number of Isolates Sequenced	Origin	Main Findings in Relation to AMR
[36]	*Aeromonas salmonicida*	101	Fish	All sequenced isolates harbored three AMR genes against beta-lactam antibiotics encoded on the chromosome. Some isolates also harbored several other plasmid encoded resistance genes against trimethoprim, sulphonamide and aminoglycoside antibiotics.
[37]	*Campylobacter* spp.	589	Retail poultry meat	The following AMR genes were identified: *tetO*, *bla*_OXA-61_, *aph(2″)-Ic*, *aph(2″)-If*, *aph(2″)-Ig*, *aph(3′)-III*, *ant(6)-1a*, *aadE*, *aph(3″)-VIIa*, and *Inu(C)*. Mutations in housekeeping genes (*gyrA* at position 86, 23S rRNA at position 2074 and 2075) associated with AMR phenotypes were also identified.
[38]	*Campylobacter* spp.	114	Humans, retail meats, and cecal samples from food production animals	Eighteen resistance genes, including *tet(O)*, *blaOXA-61*, *catA*, *lnu(C)*, *aph(2″)-Ib*, *aph(2″)-Ic*, *aph(2′)-If*, *aph(2″)-Ig*, *aph(2″)-Ih*, *aac(6′)-Ie-aph(2″)-Ia*, *aac(6′)-Ie-aph(2″)-If*, *aac(6′)-Im*, *aadE*, *sat4*, *ant(6′)*, *aad9*, *aph(3′)-Ic*, and *aph(3′)-IIIa*, and mutations in two housekeeping genes (*gyrA* and 23S rRNA), were identified.
[26]	*Campylobacter coli*	2	Retail meats	A self-transmissible plasmid carrying multiple antibiotic resistance genes was identified, carrying genes encoding resistance to gentamicin, kanamycin, streptomycin, streptothricin, and tetracycline. Gentamicin resistance was due to a phosphotransferase gene, *aph(2″)-Ig*, not described previously.
[39]	*Clostridium difficile*	40	Human and porcine origin	AMR genotypes were characterized by resistance to tetracycline [*tetM, tetA(P), tetB(P)* and *tetW*], clindamycin/erythromycin (*ermB*), and aminoglycosides (*aph3-III-Sat4A-ant6-Ia*). Resistance was mediated by clinically important mobile genetic elements, most notably Tn*6194* (harboring *ermB*) and a novel variant of Tn*5397* (harboring *tetM*).
[40]	*C. difficile*	2	Ground pork	Identification of vancomycin (*vanW, vanA, vanR, vanS, vex2, vex3, vncR, vncS*); fluoroquinolones (*gyrA* and *gyrB*); tetracyclines (*tetM*, translation elongation factor G); beta-lactams (*blaZ*); and macrolides (macrolide efflux protein, macrolide glycosyltransferase) resistance genes, and multiple multidrug resistance efflux pump genes.
[31]	*Enterococcus* spp.	197	Various animal and food sources	Resistance genotypes correlated with resistance phenotypes in 96.5% of cases for the 11 drugs investigated.
[21]	*Enterococcus faecalis, Enterococcus faecium, Escherichia coli, Salmonella enterica* serovar Typhimurium	200	Pigs	High concordance (99.74%) between phenotypic and predicted antimicrobial susceptibility was observed. Correlation between MLST type and resistance profiles was only observed in *S. enterica* serovar Typhimurium, where isolates belonging to sequence type (ST) 34 were more resistant than ST19 isolates.
[41]	ESBL-producing *Enterobacteriaceae*	24	Fish and environmental samples	Nine of eleven sequenced fish isolates had the *bla*_CTX-M-15_ gene, whereas 12/13 from environment carried *bla*_CTX-M-15_. AMR genes encoding resistance to sulfonamides (*sul1/sul2*), tetracyclines [*tet(A)/tet(B)*], fluoroquinolones [*e.g., aac(6′)-Ib-cr, qnrS1*], aminoglycosides [*e.g., aac(3)-lld, strB, strA,*] and trimethoprim (*e.g., dfrA14*) were detected.
[42]	*E. coli*	17	Retail chicken meat	All strains carried an IncK plasmid with a *bla*_CMY-2_ gene.
[43]	*E. coli*	168	Broilers and free-range retail poultry (meat/ceca)	The prevalence rates of ESBL producing *E. coli* among broiler chicken were: meat 46%; ceca 40%. Whereas, those for free range chicken were: meat 15%; ceca 30%. *E. coli* from broiler and free-range chicken exhibited varied prevalence rates for multi-drug resistance (meat 68%; ceca 64% and meat 8%; ceca 26%, respectively).
[44]	*E. coli*	18	Dairy cow manure	All sequenced isolates carried at least one β-lactamase *bla* gene: *TEM-1*, *TEM-81*, *CTX-M115*, *CTX-M15*, *OXA-1,* or *CMY-2*. Several other AMR genes were detected in the sequenced isolates and all of them harbored AMR plasmids belonging to classic Inc groups.
[45]	*E. coli*	16	Swine farm	*bla*_NDM-5_ and *mcr-1* were located on two different plasmids, which showed 100% nucleotide identity in all 16 strains.
[46]	*E. coli*	26	Humans, cows, pigs, horse, rabbit, goat, environments and food	A total of 39 plasmids were identified. Eight plasmids carried resistance genes to aminoglycosides, carbapenems, penicillins, cephalosporins, chloramphenicol, dihydrofolate reductase inhibitors, sulfonamides, tetracyclines and resistance to heavy metals. Two plasmids carried six of these resistance genes and two novel IncHI2 plasmids were also identified.
[47]	*E. coli*	42	Feedlot cattle	70% of the cattle strains carried at least one AMR gene
[48]	*E. coli*	3	Dairy cows	The *mcr-1* gene (linked to colistin resistance) coexisted with multiple resistance genes in a plasmid (pXGE1mcr)
[49]	*E. coli, Salmonella* spp.	463	Retail meats and farm local samples	To improve the concordance between genotypic and phenotypic data, it was proposed to reduce the phenotypic cut-off values for streptomycin to ≥32 µg mL(-1) for both *Salmonella* and *E. coli.*
[50]	*Helicobacter pullorum*	4	Chicken meat	AMR-associated SNPs were detected (linked to resistance to fluoroquinolones, macrolides and tetracyclines).
[51]	*H. pullorum*	11	Broiler and free-range chicken	WGS revealed the presence of five or six well characterized AMR genes, including those encoding a resistance-nodulation-division efflux pump
[30]	*Klebsiella pneumoniae*	7	Pig and human samples at abbatoirs	AMR genes associated with resistance to β-lactams, aminoglycosides, fluoroquinolones, macrolides, lincosamide, streptogramins, rifampicin, sulfonamides, trimethoprim, phenicols and tetracycline were identified.
[29]	*K. pneumoniae*	44	Chicken, turkey and pork meat	Meat-source isolates were significantly more likely to be multidrug resistant and resistant to tetracycline and gentamicin than clinical isolates. Four sequence types occurred among both meat-source and clinical isolates.
[52]	*Listeria monocytogenes*	2	Ready-to-eat food	Seven antibiotic and efflux pump related genes which may confer resistance against lincomycin, erythromycin, fosfomycin, quinolones, tetracycline, penicillin, and macrolides were identified in the genomes of both strains.
[53]	*L. monocytogenes*	5	Environments from pork processing plants	Strains of a particular sequence type were shown to contain the BAC resistance transposon Tn*6188*, conveying resistance to quaternary ammonium compounds.
[54]	*Proteus mirabilis*	8	Food-producing animals	Seven integrative and conjugative elements were identical to ICEPmiJpn1, carrying the cephalosporinase gene *bla*_CMY-2_.
[55]	Non-typhoidal *Salmonella*	536	Retail meat	A total of 65 unique resistance genes, plus mutations in two structural resistance loci, were identified. First finding of extended-spectrum β-lactamases (ESBLs) (*bla*_CTX-M1_ and *bla*_SHV2a_) in retail meat isolates of *Salmonella* in the United States.
[56]	Non-typhoidal *Salmonella*	1738	Animal, food and human sources	The Minimum Inhibitory Concentration (MIC) predictions were correlated with the ResFinder database. The genotypic cut-off values were established for 13 antimicrobials against *Salmonella*.
[20]	Non-typhoidal *Salmonella*	3491	Received by Public Health England’s Gastrointestinal Bacteria Reference Unit from different origins for surveillance purposes	Discrepancies between phenotypic and genotypic profiles for one or more antimicrobials were detected for 76 isolates (2.18%). Only 88/52,365 (0.17%) isolate/antimicrobial combinations were discordant. Pan-susceptibility to antimicrobials was observed in 2190 isolates (62.73%).
[33]	*S. enterica*	90	Dairy cattle and humans	Genotypic prediction of phenotypic resistance resulted in a mean sensitivity of 97.2 and specificity of 85.2.
[57]	*S.enterica* serovar *Typhimurium*	984	Swine	Multiple genotypic resistance determinants were predominant, including resistance against ampicillin, streptomycin, sulfonamides, and tetracyclines. Phenotypic resistance to enrofloxacin and ceftiofur was found in conjunction with the presence of plasmid-mediated AMR genes.
[58]	*S.enterica* serovar Typhimurium	1	Swine carcass	The following AMR genes were identified: *tetA*, *aac3IIa, aadA1, strA, strB*, *bla*_TEM-1B_, *qnrE, sul1, drfA1,* and *floR*.
[33]	*S. enterica*	90	Dairy cattle and humans	WGS-based prediction of phenotypic resistance resulted in a mean sensitivity of 97.2 and specificity of 85.2.
[59]	*S.enterica* serovar Heidelberg	113	Humans, abbatoir poultry and retail poultry	CMY-2 plasmids, all belonging to incompatibility group I1, were identified in cefoxitin-resistant isolates. Analysis of IncI1 plasmid sequences revealed high identity (95 to 99%) to a previously described plasmid (pCVM29188_101) found in *S. enterica* serovar Kentucky.
[60]	*S.enterica* serovar Indiana	1	Poultry slaughterhouse	24 multi-drug resistance (MDR) genes, located on 4 plasmids, were identified, including the *mcr-1* gene (linked to colistin resistance).
[61]	*S.enterica* serovar Infantis	12	Humans, food-producing animals and meat	Some isolates harbored a conjugative megaplasmid (~280–320 Kb) which carried the ESBL gene *blaCTX-M-1*, and additional genes [*tet(A), sul1, dfrA1* and *dfrA14*] mediating cefotaxime, tetracycline, sulfonamide, and trimethoprim resistance.
[62]	*S.enterica* serovar Muenster	2	Dairy farm environments	The plasmid-mediated *qnrB19* gene and IncR plasmid type were identified in both isolates.
[63]	*S.enterica* serovar Typhimurium	225	Humans, animals, feed, and food	The non-clinical use of narrow-spectrum penicillins (e.g., benzylpenicillin) might have favoured the diffusion of plasmids carrying the *bla_TEM-1_* gene in *S. enterica* serotype Typhimurium in the late 1950s.
[64]	*S.enterica* serovar Typhimurium	4	Poultry and humans	The following AMR genes were identified: *strA*, *strB*, and *aadA1* (aminoglycosides); *bla*_TEM-1B_ (β-lactams); *catA1* (phenicols); *sul1* and *sul2* (sulphonamides); *tet B* (tetracyclines); and *dfrA1* (trimethoprim).
[65]	*S.enterica* serovar Typhimurium and *S.* *enterica* serovar Kentucky	2	Chicken carcasses	A total of five plasmids conveying AMR genes were found.
[66]	*S.enterica* serovar Weltevreden	44	Human stool and contaminated food samples	AMR genes were only identified in eight isolates, linked to resistance to tetracycline, ciprofloxacin or ampicillin.
[67]	*Staphylococcus aureus*	66	Retail meats	Eleven *spa* types were represented. The majority of MRSA (84.8%) possessed SCC*mec* IV.
[68]	*S. aureus*	9	Pork, chicken and turkey meat	Multiple resistance genes/mutations were detected. All livestock-associated methicillin-resistant *S. aureus* (LA-MRSA) harbored *tet(M)* (±*tet(K)* and *tet(L)*), and only seven of these additionally harbored multi-drug resistance to beta-lactams, quinolones and macrolides.
[69]	*S. aureus*	12	Livestock animals	Most isolates harbored resistance genes to ≥3 antimicrobial classes in addition to β-lactams. Heavy metal resistance genes were detected in most European *ccrC* positive isolates, with >80% harboring *czrC*, encoding zinc and cadmium resistance.
[70]	*S. aureus*	15	Bulk milk	A divergent *mec*A homologue (*mec*A_LGA251_), later named as *mec*C, was identified.
[8]	*Streptococcus thermophilus*	5	Raw milk	*tet(S)* and *ermB* identified as determinants of AMR.
[71]	Carbapenem-resistant bacteria	28	Dairy cattle	Isolates included: 3 *E. coli* harbouring *bla*_CMY-2_ and truncated *ompF* genes; 8 *Aeromonas* harbouring *bla*_cphA_-like genes; 1 *Acinetobacter baumannii* harbouring a novel *bla*_OXA_ gene (*bla*_OXA-497_); and 6 *Pseudomonas* with conserved domains of various carbapenemase-producing genes.
