# Correction: Oniciuc, E. A.; et al. The Present and Future of Whole Genome Sequencing (WGS) and Whole Metagenome Sequencing (WMS) for Surveillance of Antimicrobial Resistant Microorganisms and Antimicrobial Resistance Genes across the Food Chain. *Genes* 2018, *9*, 268.

**DOI:** 10.3390/genes9070315

**Published:** 2018-06-25

**Authors:** Elena A. Oniciuc, Eleni Likotrafiti, Adrián Alvarez-Molina, Miguel Prieto, Jesús A. Santos, Avelino Alvarez-Ordóñez

**Affiliations:** 1 Faculty of Food Science and Engineering, Dunarea de Jos University of Galati, Galati 800008, Romania; elena.oniciuc@ugal.ro; 2 Laboratory of Food Microbiology, Department of Food Technology, Alexander Technological Educational Institute of Thessaloniki, Thessaloniki T.K. 57400, Greece; likotraf@food.teithe.gr; 3 Department of Food Hygiene and Technology and Institute of Food Science and Technology, Universidad de León, 24071 León, Spain; aalvm@unileon.es (A.A.-M.); miguel.prieto@unileon.es (M.P.); j.santos@unileon.es (J.A.S.)

The authors wish to make the following changes to their paper [1]. Due to an undetected mistake in the references management, certain errors appeared in the reference list and a reference was duplicated in Table 1. Consequently, three references have been changed as follows:

Reference [51] “51. Kumar, N.; Mariappan, V.; Baddam, R.; Lankapalli, A.K.; Shaik, S.; Goh, K.L.; Loke, M.F.; Perkins, T.; Benghezal, M.; Hasnain, S.E.; et al. Comparative Genomic Analysis of *Helicobacter pylori* from Malaysia Identifies Three Distinct Lineages Suggestive of Differential Evolution. *Nucleic Acids Res.* **2015**, *43*, 324–335.” has been replaced by: “51. Qumar, S.; Majid, M.; Kumar, N.; Tiwari, S.K.; Semmler, T.; Devi, S.; Baddam, R.; Hussain, A.; Shaik, S.; Ahmed, N. Genome Dynamics and Molecular Infection Epidemiology of Multidrug-Resistant *Helicobacter pullorum* Isolates Obtained from Broiler and Free-Range Chickens in India. *Appl. Environ. Microbiol*. **2017**, *83*, e02305-16.”

Reference [44] “44. Rehman, M.U.; Zhang, H.; Iqbal, M.K.; Mehmood, K.; Huang, S.; Nabi, F.; Luo, H.; Lan, Y.; Li, J. Antibiotic Resistance, Serogroups, Virulence Genes, and Phylogenetic Groups of *Escherichia coli* Isolated from Yaks with Diarrhea in Qinghai Plateau, China. Gut Pathog. **2017**, *9*, 1–11.” has been replaced by “44. Rehman, M.A.; Yin, X.; Lepp, D.; Laing, C.; Ziebell, K.; Talbot, G.; Topp, E.; Diarra, M.S. Genomic Analysis of Third Generation Cephalosporin Resistant *Escherichia coli* from Dairy Cow Manure. *Vet. Sci.* **2017**, *4*, 4, doi:10.3390/vetsci4040057.”

Reference [60] “60. Wang, W.; Baloch, Z.; Peng, Z.; Hu, Y.; Xu, J.; Fanning, S.; Li, F. Genomic Characterization of a Large Plasmid Containing a *bla_NDM-1_* Gene Carried on *Salmonella enterica* Serovar Indiana C629 Isolate from China. *BMC Infect. Dis.* **2017**, *17*, 1–8.” has been replaced by “60. Wang, J.; Li, X.; Li, J.; Hurley, D.; Bai, X.; Yu, Z.; Cao, Y.; Wall, E.; Fanning, S.; Bai, L. Complete Genetic Analysis of a *Salmonella enterica* serovar Indiana Isolate Accompanying Four Plasmids Carrying *mcr-1*, ESBL and Other Resistance Genes in China. *Vet. Microbiol*. **2017**, *210*, 142–146, doi:10.1016/j.vetmic.2017.08.024.”

Two references have been added, as they were omitted in error:

Reference [66] in Table 1 was wrongly cited, therefore it has been substituted by the new reference [66] “66. Li, B.; Yang, X.; Tan, H.; Ke, B.; He, D.; Wang, H.; Chen, Q.; Ke, C.; Zhang, Y. Whole Genome Sequencing Analysis of *Salmonella enterica* Serovar Weltevreden Isolated from Human Stool and Contaminated Food Samples Collected from the Southern Coastal Area of China. *Int. J. Food Microbiol.* **2018**, *266*, 317–323, doi:10.1016/j.ijfoodmicro.2017.10.032.”

Reference [8] in Table 1 was wrongly cited, therefore it has been substituted by the new reference [71] “71. Flórez, A.B.; Mayo, B. Antibiotic Resistance-Susceptibility Profiles of *Streptococcus thermophilus* Isolated from Raw Milk and Genome Analysis of the Genetic Basis of Acquired Resistances. *Front. Microbiol.* **2017**, *8*, 2608, doi:10.3389/fmicb.2017.02608.”

Due to this correction, reference numbers were adjusted to follow a numerical order. In [1], the previous references [66] and [71] are now [139] and [72], respectively.

The 31st row from Table 1, about *S. enterica* with origin in Dairy cattle and humans, was eliminated because it was a duplicate of row 28; the corrected table is:

The authors would like to apologize for any inconvenience caused to the readers by these changes.

## Figures and Tables

**Table 1:** Main research studies published in recent years applying whole genome sequencing (WGS) to characterize antimicrobial resistance (AMR) in foodborne bacteria.

Reference	Microbial Species	Number of Isolates Sequenced	Origin	Main Findings in Relation to AMR
[36]	*Aeromonas salmonicida*	101	Fish	All sequenced isolates harbored three AMR genes against beta-lactam antibiotics encoded on the chromosome. Some isolates also harbored several other plasmid encoded resistance genes against trimethoprim, sulphonamide, and aminoglycoside antibiotics.
[37]	*Campylobacter* spp*.*	589	Retail poultry meat	The following AMR genes were identified: *tetO*, *bla*_OXA-61_, *aph(2″)-Ic*, *aph(2″)-If*, *aph(2″)-Ig*, *aph(3′)-III*, *ant(6)-1a*, *aadE*, *aph(3″)-VIIa*, and *Inu(C)*. Mutations in housekeeping genes (*gyrA* at position 86, 23S rRNA at position 2074 and 2075) associated with AMR phenotypes were also identified.
[38]	*Campylobacter* spp.	114	Humans, retail meats, and cecal samples from food production animals	Eighteen resistance genes, including *tet(O)*, *blaOXA-61*, *catA*, *lnu(C)*, *aph(2″)-Ib*, *aph(2″)-Ic*, *aph(2′)-If*, *aph(2″)-Ig*, *aph(2″)-Ih*, *aac(6′)-Ie-aph(2″)-Ia*, *aac(6′)-Ie-aph(2″)-If*, *aac(6′)-Im*, *aadE*, *sat4*, *ant(6′)*, *aad9*, *aph(3′)-Ic*, and *aph(3′)-IIIa*, and mutations in two housekeeping genes (*gyrA* and 23S rRNA), were identified.
[26]	*Campylobacter coli*	2	Retail meats	A self-transmissible plasmid carrying multiple antibiotic resistance genes was identified, carrying genes encoding resistance to gentamicin, kanamycin, streptomycin, streptothricin, and tetracycline. Gentamicin resistance was due to a phosphotransferase gene, *aph(2″)-Ig*, not described previously.
[39]	*Clostridium difficile*	40	Human and porcine origin	AMR genotypes were characterized by resistance to tetracycline [*tetM*, *tetA(P)*, *tetB(P)*, and *tetW*], clindamycin/erythromycin (*ermB*), and aminoglycosides (*aph3-III-Sat4A-ant6-Ia*). Resistance was mediated by clinically important mobile genetic elements, most notably Tn*6194* (harboring *ermB*) and a novel variant of Tn*5397* (harboring *tetM*).
[40]	*C. difficile*	2	Ground pork	Identification of vancomycin (*vanW*, *vanA*, *vanR*, *vanS*, *vex2*, *vex3*, *vncR*, *vncS*); fluoroquinolones (*gyrA* and *gyrB*); tetracyclines (*tetM*, translation elongation factor G); beta-lactams (*blaZ*); and macrolides (macrolide efflux protein, macrolide glycosyltransferase) resistance genes, and multiple multidrug resistance efflux pump genes.
[31]	*Enterococcus* spp.	197	Various animal and food sources	Resistance genotypes correlated with resistance phenotypes in 96.5% of cases for the 11 drugs investigated.
[21]	*Enterococcus faecalis*, *Enterococcus faecium*, *Escherichia coli*, *Salmonella enterica* serovar Typhimurium	200	Pigs	High concordance (99.74%) between phenotypic and predicted antimicrobial susceptibility was observed. Correlation between MLST type and resistance profiles was only observed in *S. enterica* serovar Typhimurium, where isolates belonging to sequence type (ST) 34 were more resistant than ST19 isolates.
[41]	ESBL-producing *Enterobacteriaceae*	24	Fish and environmental samples	Nine of eleven sequenced fish isolates had the *bla*_CTX-M-15_ gene, whereas 12/13 from environment carried *bla*_CTX-M-15_*.* AMR genes encoding resistance to sulfonamides (*sul1*/*sul2*), tetracyclines [*tet(A)/tet(B)*], fluoroquinolones [e.g., *aac(6′)-Ib-cr*, *qnrS1*], aminoglycosides [e.g., *aac(3)-lld*, *strB*, *strA*], and trimethoprim (e.g., *dfrA14*) were detected.
[42]	*E. coli*	17	Retail chicken meat	All strains carried an IncK plasmid with a *bla*_CMY-2_ gene.
[43]	*E. coli*	168	Broilers and free-range retail poultry (meat/ceca)	The prevalence rates of ESBL producing *E. coli* among broiler chicken were: meat 46%; ceca 40%. Whereas, those for free range chicken were: meat 15%; ceca 30%. *E. coli* from broiler and free-range chicken exhibited varied prevalence rates for multi-drug resistance (meat 68%; ceca 64% and meat 8%; ceca 26%, respectively).
[44]	*E. coli*	18	Dairy cow manure	All sequenced isolates carried at least one β-lactamase *bla* gene: *TEM-1*, *TEM-81*, *CTX-M115*, *CTX-M15*, *OXA-1*, or *CMY-2*. Several other AMR genes were detected in the sequenced isolates and all of them harbored AMR plasmids belonging to classic Inc groups.
[45]	*E. coli*	16	Swine farm	*bla*_NDM-5_ and *mcr-1* were located on two different plasmids, which showed 100% nucleotide identity in all 16 strains.
[46]	*E. coli*	26	Humans, cows, pigs, horse, rabbit, goat, environments and food	A total of 39 plasmids were identified. Eight plasmids carried resistance genes to aminoglycosides, carbapenems, penicillins, cephalosporins, chloramphenicol, dihydrofolate reductase inhibitors, sulfonamides, tetracyclines, and resistance to heavy metals. Two plasmids carried six of these resistance genes and two novel IncHI2 plasmids were also identified.
[47]	*E. coli*	42	Feedlot cattle	70% of the cattle strains carried at least one AMR gene
[48]	*E. coli*	3	Dairy cows	The *mcr-1* gene (linked to colistin resistance) coexisted with multiple resistance genes in a plasmid (pXGE1mcr)
[49]	*E. coli*, *Salmonella* spp.	463	Retail meats and farm local samples	To improve the concordance between genotypic and phenotypic data, it was proposed to reduce the phenotypic cut-off values for streptomycin to ≥32 µg mL^−1^ for both *Salmonella* and *E. coli.*
[50]	*Helicobacter pullorum*	4	Chicken meat	AMR-associated SNPs were detected (linked to resistance to fluoroquinolones, macrolides, and tetracyclines).
[51]	*H. pullorum*	11	Broiler and free-range chicken	WGS revealed the presence of five or six well characterized AMR genes, including those encoding a resistance-nodulation-division efflux pump
[30]	*Klebsiella pneumoniae*	7	Pig and human samples at abbatoirs	AMR genes associated with resistance to β-lactams, aminoglycosides, fluoroquinolones, macrolides, lincosamide, streptogramins, rifampicin, sulfonamides, trimethoprim, phenicols, and tetracycline were identified.
[29]	*K. pneumoniae*	44	Chicken, turkey and pork meat	Meat-source isolates were significantly more likely to be multidrug resistant and resistant to tetracycline and gentamicin than clinical isolates. Four sequence types occurred among both meat-source and clinical isolates.
[52]	*Listeria monocytogenes*	2	Ready-to-eat food	Seven antibiotic and efflux pump related genes which may confer resistance against lincomycin, erythromycin, fosfomycin, quinolones, tetracycline, penicillin, and macrolides were identified in the genomes of both strains.
[53]	*L. monocytogenes*	5	Environments from pork processing plants	Strains of a particular sequence type were shown to contain the BAC resistance transposon Tn*6188*, conveying resistance to quaternary ammonium compounds.
[54]	*Proteus mirabilis*	8	Food-producing animals	Seven integrative and conjugative elements were identical to ICEPmiJpn1, carrying the cephalosporinase gene *bla*_CMY-2_.
[55]	Non-typhoidal *Salmonella*	536	Retail meat	A total of 65 unique resistance genes, plus mutations in two structural resistance loci, were identified. First finding of extended-spectrum β-lactamases (ESBLs) (*bla*_CTX-M1_ and *bla*_SHV2a_) in retail meat isolates of *Salmonella* in the United States.
[56]	Non-typhoidal *Salmonella*	1738	Animal, food and human sources	The Minimum Inhibitory Concentration (MIC) predictions were correlated with the ResFinder database. The genotypic cut-off values were established for 13 antimicrobials against *Salmonella*.
[20]	Non-typhoidal *Salmonella*	3491	Received by Public Health England’s Gastrointestinal Bacteria Reference Unit from different origins for surveillance purposes	Discrepancies between phenotypic and genotypic profiles for one or more antimicrobials were detected for 76 isolates (2.18%). Only 88/52,365 (0.17%) isolate/antimicrobial combinations were discordant. Pan-susceptibility to antimicrobials was observed in 2190 isolates (62.73%).
[33]	*S. enterica*	90	Dairy cattle and humans	Genotypic prediction of phenotypic resistance resulted in a mean sensitivity of 97.2 and specificity of 85.2.
[57]	*S.* *enterica* serovar *Typhimurium*	984	Swine	Multiple genotypic resistance determinants were predominant, including resistance against ampicillin, streptomycin, sulfonamides, and tetracyclines. Phenotypic resistance to enrofloxacin and ceftiofur was found in conjunction with the presence of plasmid-mediated AMR genes.
[58]	*S.* *enterica* serovar Typhimurium	1	Swine carcass	The following AMR genes were identified: *tetA*, *aac3IIa*, *aadA1*, *strA*, *strB*, *bla*_TEM-1B_, *qnrE*, *sul1*, *drfA1*, and *floR*.
[59]	*S.* *enterica* serovar Heidelberg	113	Humans, abbatoir poultry and retail poultry	CMY-2 plasmids, all belonging to incompatibility group I1, were identified in cefoxitin-resistant isolates. Analysis of IncI1 plasmid sequences revealed high identity (95% to 99%) to a previously described plasmid (pCVM29188_101) found in *S. enterica* serovar Kentucky*.*
[60]	*S.* *enterica* serovar Indiana	1	Poultry slaughterhouse	24 multi-drug resistance (MDR) genes, located on 4 plasmids, were identified, including the *mcr-1* gene (linked to colistin resistance).
[61]	*S.* *enterica* serovar Infantis	12	Humans, food-producing animals and meat	Some isolates harbored a conjugative megaplasmid (~280–320 Kb) which carried the ESBL gene *blaCTX-M-1*, and additional genes [*tet(A)*, *sul1*, *dfrA1* and *dfrA14*] mediating cefotaxime, tetracycline, sulfonamide, and trimethoprim resistance.
[62]	*S.* *enterica* serovar Muenster	2	Dairy farm environments	The plasmid-mediated *qnrB19* gene and IncR plasmid type were identified in both isolates.
[63]	*S.* *enterica* serovar Typhimurium	225	Humans, animals, feed, and food	The non-clinical use of narrow-spectrum penicillins (e.g., benzylpenicillin) might have favored the diffusion of plasmids carrying the *bla_TEM-1_* gene in *S. enterica* serotype Typhimurium in the late 1950s.
[64]	*S.* *enterica* serovar Typhimurium	4	Poultry and humans	The following AMR genes were identified: *strA*, *strB*, and *aadA1* (aminoglycosides); *bla*_TEM-1B_ (β-lactams); *catA1* (phenicols); *sul1* and *sul2* (sulphonamides); *tet B* (tetracyclines); and *dfrA1* (trimethoprim)*.*
[65]	*S.* *enterica* serovar Typhimurium and *S.* *enterica* serovar Kentucky	2	Chicken carcasses	A total of five plasmids conveying AMR genes were found.
[66]	*S.* *enterica* serovar Weltevreden	44	Human stool and contaminated food samples	AMR genes were only identified in eight isolates, linked to resistance to tetracycline, ciprofloxacin or ampicillin.
[67]	*Staphylococcus aureus*	66	Retail meats	Eleven *spa* types were represented. The majority of MRSA (84.8%) possessed SCC*mec* IV.
[68]	*S. aureus*	9	Pork, chicken and turkey meat	Multiple resistance genes/mutations were detected. All livestock-associated methicillin-resistant *S. aureus* (LA-MRSA) harbored *tet(M)* (±*tet(K)* and *tet(L)*), and only seven of these additionally harbored multi-drug resistance to beta-lactams, quinolones, and macrolides.
[69]	*S. aureus*	12	Livestock animals	Most isolates harbored resistance genes to ≥3 antimicrobial classes in addition to β-lactams. Heavy metal resistance genes were detected in most European *ccrC* positive isolates, with >80% harboring *czrC*, encoding zinc and cadmium resistance.
[70]	*S. aureus*	15	Bulk milk	A divergent *mec*A homologue (*mec*A_LGA251_), later named as *mec*C, was identified.
[71]	*Streptococcus thermophilus*	5	Raw milk	*tet(S)* and *ermB* identified as determinants of AMR.
[72]	Carbapenem-resistant bacteria	28	Dairy cattle	Isolates included: 3 *E. coli* harboring *bla*_CMY-2_ and truncated *ompF* genes; 8 *Aeromonas* harboring *bla*_cphA_-like genes; 1 *Acinetobacter baumannii* harboring a novel *bla*_OXA_ gene (*bla*_OXA-497_); and 6 *Pseudomonas* with conserved domains of various carbapenemase-producing genes.
